# Enterovirus 71-Associated Infection in South Vietnam: Vaccination Is a Real Solution

**DOI:** 10.3390/vaccines11050931

**Published:** 2023-05-03

**Authors:** Natalia I. Romanenkova, Thi Thanh Thao Nguyen, Liudmila N. Golitsyna, Natalia V. Ponomareva, Nadezhda R. Rozaeva, Olga I. Kanaeva, Artem V. Leonov, Nadezhda A. Novikova, Maina A. Bichurina

**Affiliations:** 1 Saint Petersburg Pasteur Institute, St. Petersburg 197101, Russiakanaeva@pasteurorg.ru (O.I.K.);; 2 Pasteur Institute, Ho Chi Minh City 167 Pasteur, Phường Võ Thị Sáu, Quận 3, TP. Hồ Chí Minh 643103, Vietnam; 3 Blokhina Nizhny Novgorod Research Institute of Epidemiology and Microbiology, Nizhny Novgorod 603950, Russia

**Keywords:** hand-foot-and-mouth disease, enteroviral meningitis, acute flaccid paralysis, enterovirus 71, circulation, prevention, non-polio enterovirus 71 vaccine

## Abstract

Hand-foot-and-mouth disease (HFMD) is the most common enteroviral infection in South-East Asia. When evaluating the role of enterovirus 71 (EVA71) as an etiological agent of infectious disease in South Vietnam, we revealed a high proportion of EVA71 among identified species A enteroviruses found in 3542 samples from HFMD cases; 125 samples from cases of enteroviral meningitis; and 130 samples from acute flaccid paralysis (AFP) cases. These represent 50%, 54.8%, and 51.5%, respectively. According to molecular analysis, 90% of EVA71 were attributed to genotype C4 and 10% were attributed to genotype B5. The predominance of EVA71 circulation among the population proves the need to strengthen surveillance (with monitoring of enterovirus circulation for facilitation of HFMD outbreak prediction) and to increase the effectiveness of preventative measures by the implementation of vaccination against EVA71-associated infections. A phase III trial of a Taiwanese vaccine (EV71vac) in Taiwan and South Vietnam showed its safety, tolerability, and efficacy in children aged 2–71 months. This B4 genotype-based vaccine, which features cross-protection against B5 and C4 genotypes, and other existing EV71 vaccines can serve as a good approach to solving the HFMD problem, which is so important for Vietnam.

## 1. Introduction

Enteroviruses A-D species genus Enteroviruses family Picornaviridae are associated with human enteroviral infection. Enteroviral infection (EVI) is characterized by a wide diversity of clinical forms, including enteroviral meningitis, meningoencephalitis, hand-foot-and-mouth disease (HFMD), and other clinical manifestations [1,2,3,4,5,6,7,8,9,10,11,12].

In Vietnam, the first cases of hand-foot-and-mouth disease complicated by encephalitis were reported during an encephalitis outbreak in 2003 when enterovirus EVA71 was isolated by researchers of the Pasteur Institute in Ho Chi Minh City as one of the etiological agents of the disease. The epidemiological and virological data of this outbreak were presented in some publications [9,13]. During a sharp increase in HFMD incidence in Vietnam, 3791 children were admitted to the Ho Chi Minh Children’s Hospital (between September and November 2011) with symptoms of HFMD. Severe disease was observed in 12% of patients, and six children died. Among the enteroviruses detected in patients with severe forms, 76% were EVA71 viruses [13]. In Ho Chi Minh City, the average age of patients was 20 months; 93.4% of cases were children under 3 years of age. The southern provinces of Vietnam had the largest proportion of cases and deaths.

HFMD can vary in clinical manifestation and severity: from mild febrile illness to serious, multisystemic disease accompanied by damage to the cardiovascular and central nervous systems [11]. Complications can lead to cognitive and motor disorders. Death due to pulmonary edema or encephalitis is possible. Lethality ranges from 0.5% to 19.0%, depending on the viral genotype [14,15].

The system of continuous HFMD surveillance was created in Vietnam after the classification of HFMD as a disease with high epidemic potential, and in 2011, monitoring and weekly reporting of HFMD cases were installed by the Vietnamese Ministry of Health [7]. Obligatory laboratory confirmation of severe and/or fatal cases and identification of isolated enteroviral strains are required.

## 2. Materials and Methods

### 2.1. Study Design

The study was undertaken within a framework of scientific and technical cooperation between research institutes in Russia and Vietnam. The collaborative research performed from 2018 to 2022 was approved by the ethical committees of three institutes (the Saint-Petersburg Pasteur Institute, the Blokhina Research Institute of Epidemiology and Microbiology (Nizhny Novgorod), and the Pasteur Institute in Ho Chi Minh City). A comparative analysis of data obtained from 2010–2021 during long-term surveillance of enteroviral infections was done. During this period, the fecal samples from children treated in the main provincial hospitals of South Vietnam were sent to the Pasteur Institute in Ho Chi Minh City, where virus isolation and/or PCR were done. The final clinical diagnosis was determined by clinicians from the hospitals. In 2018, part of the material was delivered to Russia, where the virus type determination and complete molecular investigations were carried out in 2018–2022. In this period, the analysis of the results obtained in different periods of time was also done. Cases of HFMD, meningitis, encephalitis, or acute flaccid paralysis (AFP) were diagnosed and virologically investigated according to WHO recommendations [16,17]. Study participants were hospitalized children from South Vietnam with suspected HFMD, AFP, or with other symptoms of enteroviral infection. Parental informed consent was received for all hospitalized patients according to the requirements of the Vietnamese Ministry of Public Health.

### 2.2. Virological and Serological Methods

Isolation and identification of viruses from fecal samples were performed by standard WHO procedures [16]. Viruses were isolated using RD, L20B, Hep-2c, and Vero cell lines [17]. Virus identification was performed in a neutralization assay with specific diagnostic sera on the cell culture in which the viral strain was isolated in accordance with WHO recommendations [16]. ITD of polioviruses was carried out using ELISA with polyclonal cross-adsorbed sera, real-time RT-PCR with specific primers, and neutralization with monoclonal antibodies to wild and vaccine polioviruses [18,19].

### 2.3. Molecular Methods

Identification of enteroviral type was performed by RT-PCR and partial sequencing of the VP1 genomic region, using oligonucleotide primers and a protocol developed by Nix et al. [20]. Determination of the complete VP1 genomic region sequence of EV-A71 was carried out with the help of species-specific RT-PCR amplification [21]. RNA was extracted from viral isolates with a QIAamp Viral RNA mini kit (QIAGEN, Inc.). Amplified cDNA fragments were sequenced using the GenomeLab DTCS—Quick Start Kit on a GenomeLab ™ GeXP automated genetic analyzer (Beckman Coulter, USA). Alignment of nucleotide sequences, construction of phylogenic trees, and analysis of phylogenetic relationships were performed using MEGA 7.0 software [22]. Sequences of Vietnamese EV71 strains and sequences available in the GenBank database were used for analysis. Nucleotide sequence alignments were performed using ClustalW. A phylogenetic tree for EV-A71 VP1 coding sequences (891 nt) was constructed using the maximum likelihood method based on the Tamura–Nei model and validated by bootstrapping with 1000 replicates.

### 2.4. Statistical Analysis

The average errors were determined, and the significance of statistical differences was assessed using the Student’s *t*-test. Differences were considered statistically significant using a 95% confidence interval (values of *p* < 0.05).

## 3. Results

### 3.1. Analysis of Long-Term Virological Surveillance of Different Clinical Forms of Enteroviral Infection

In South Vietnam, continuous surveillance of different clinical forms of enteroviral infection was performed according to the national system, as was the surveillance for acute flaccid paralysis (executed within the framework of the Global Polio Eradication Initiative). From 2012 to 2021, in 20 southern provinces of Vietnam, 12,798 hospitalized HFMD patients were investigated. In 3610 cases, strains were identified, and enteroviral types were determined. The amount of species B enteroviruses was only 67 (1.8%), one enterovirus belonged to species C (CVA24), and the other 3542 (98.2%) enteroviruses were classified into species A. Half of the species A (1771 strains) enteroviruses were represented by EVA71 virus, and the other half belonged to seven types of CVA viruses, with a dominance of three types (CVA6—830 strains, CVA10—359 strains, CVA16—255 strains). Their percentage was 81.5% of all isolated CVA viruses.

When analyzing clinical data from all involved hospitals in South Vietnam, 54.7% of HFMD cases studied were found to be complicated. As a rule, the etiological agent was enterovirus 71 in such cases. According to a detailed clinical analysis of HFMD case severity in three selected hospitals in the southern provinces, the picture was similar. In 75% of patients who had complicated forms of the disease, we isolated enterovirus 71 as the leading type among all HFMD etiological agents.

It should be noted that enteroviruses of species A were revealed not only in patients with HFMD but also in patients with enteroviral meningitis or meningoencephalitis and in children with the syndrome of acute flaccid paralysis. For the 2012–2021 period, 228 strains of non-polio enteroviruses were identified in patients with a diagnosis of enteroviral meningitis or meningoencephalitis. Species A enteroviruses constituted 57%, and species B enteroviruses constituted 43% of revealed viruses. One EVD68 virus was also identified. Among species A viruses, enterovirus 71 prevailed. Its share was 73.8%. CVA6 virus took second place and constituted 10%.

From 2010–2021, 22 polioviruses were isolated from 2143 AFP patients and identified. Of these strains, 20 were vaccine polioviruses, and two strains, isolated in 2012, were type 2 VDPV. In the AFP patient’s samples, some of whom had a primary diagnosis of acute flaccid myelitis, 239 strains of non-polio enteroviruses were also isolated and identified. The number of enteroviruses of species A (125 strains) and species B (112 strains) was approximately equal; two strains were identified as CVA21 and CVA24 (species C). More than half of species A viruses (67 strains, 53.6%) belonged to the enterovirus circulating predominantly in Vietnam, EVA71. The tiers of isolated viruses were represented by two types: 24 CVA10 (19.2%) and 17 CVA16 (13.6%). In total, we revealed a high proportion of EVA71 strains among identified species A enteroviruses that were found in 3542 samples from patients with HFMD, in 125 samples from cases of meningitis, and in 125 samples from patients with AFP. These represented 50%, 54.8%, and 51.5%, respectively.

**Figure 1:**
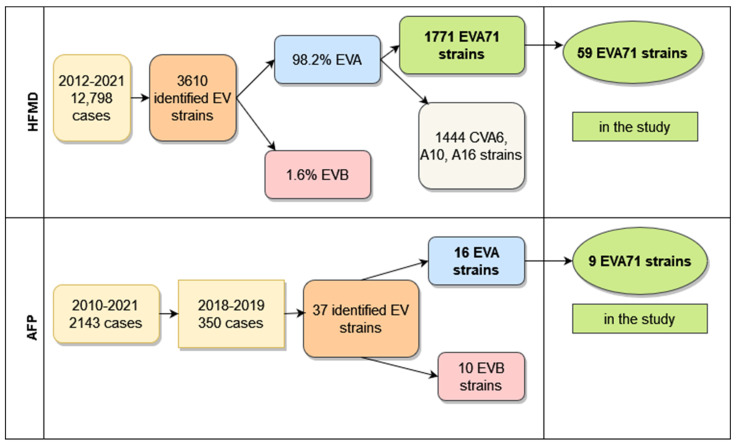
Total amount of HFMD and AFP cases in South Vietnam.

Figure 1 represents the total amount of EVI cases with different clinical forms investigated during long-term surveillance and the number of identified enteroviral strains from these cases.

### 3.2. Non-Polio Enteroviruses Isolated from Hand-Foot-and-Mouth Disease Cases

In this collaborative study, we revealed 113 non-polio enteroviruses isolated or identified in samples of patients suffering from HFMD (87) or children with a diagnosis of acute flaccid paralysis (26). All the studied HFMD cases were associated with species A enteroviruses, and the majority of them (59 strains, 67.8%) belonged to type EVA71. The others (32.2%) were represented by four other types of species A enteroviruses: 20 CVA10, 5 CVA16, 2 CVA6, and 1 CVA2. Table 1 shows clinical and epidemiological data of patients with HFMD from whom species A enteroviruses were isolated.

It is important to emphasize that most HFMD cases were severe and characterized by troubles in the neurological, cardiovascular, and respiratory systems. The share of complicated clinical forms was 86.2%, and it significantly exceeded (*p* < 0.001) the percentage of mild clinical forms of the disease (13.8%), wherein papulovesicular rash (hands, feet, oral mucosa) was accompanied by normal or sub-febrile temperature. The high share of complicated clinical forms was connected with the predominance of EVA71-associated cases of HFMD (70.7%). In cases of complicated HFMD, in addition to the typical rash, patients had a high fever and numerous neurological or cardiovascular manifestations, such as muscle cramps, confusion, meningitis, or myocarditis. In eight patients with maximum illness severity, severe complications involving the nervous, cardiovascular, and respiratory systems were noted. In some patients, paralytic manifestations were also noted. In one case, we recorded a fatal outcome. The child, aged 19 months, who had multiple disorders, died from toxic shock and pulmonary edema. The majority of patients (88.5%) suffering from HFMD were younger than three years old. The share of such children significantly exceeded (*p* < 0.001) the proportion of children of 3–6 years of age. The percentage of boys exceeded (*p* < 0.05) the same indicator for girls (62.1% and 37.9% consequently).

Epidemiological analysis of 59 HFMD cases associated with EVA71 showed that 78% of them were registered in three provinces located in the Mekong Delta, predominantly in the province of Dong Thap, bordering the Kingdom of Cambodia. This province, together with the neighboring province of An Giang, represented a single epidemiological focus of HFMD.

### 3.3. Non-Polio Enteroviruses Isolated from Acute Flaccid Paralysis Cases

In 2018–2019, we isolated 37 non-polio enteroviruses from 350 AFP cases (10.6%). Half of them belonged to species A, and half to species B. Identification of 26 non-polio enteroviruses isolated from AFP cases, which were studied in cooperation, showed that 16 of them belonged to species A (8 strains were identified as EVA71, 6 strains as CVA10, and 2 strains as CVA16), and 10 strains were enteroviruses of species B (1 CVA9, 2 CVB2, 2 Echo6, 3 Echo11, 2 Echo20). All the children (Table 2) had a typical clinical picture of AFP syndrome. Most children (69.2%) were less than three years of age; 30.8% of children were over 3 years old. Among those with AFP, the share of boys (65.4%) exceeded (*p* < 0.05) the share of girls (34.6%).

### 3.4. Comparative Analysis of EVA71 Strains Isolated from Patients with HFMD and AFP

**Figure 2:**
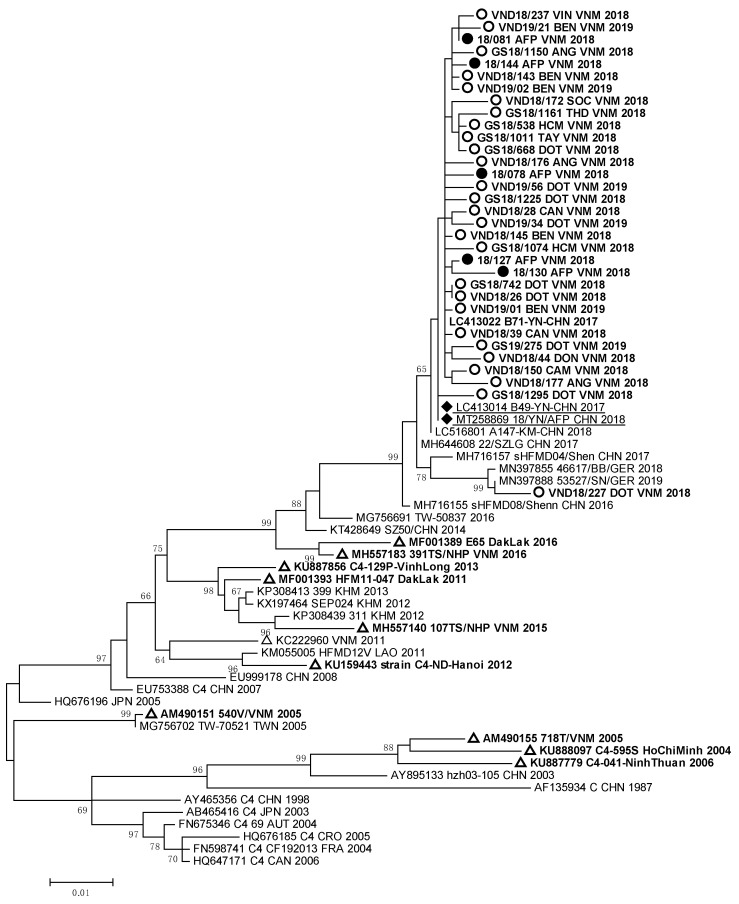
Phylogenic relationships between EVA71 strains of genotype C4 identified in Vietnam: ○—Vietnamese strains isolated from HFMD patients; ●—Vietnamese strains isolated from AFP patients; ∆—Vietnamese strains described in other studies; ♦—strains identified in patients with AFP from the Chinese province of Yunnan.

Molecular analysis showed that 88% of EVA71 strains isolated from HFMD and AFP patients in South Vietnam belonged to genotype C4, and 12% of the EVA71 strains were represented by genotype B5. The full nucleotide sequences of the VP1 genomic region of 67 EVA71 viruses were submitted to GenBank under the numbers MW139687–MW139744 and OP947996–OP948003. Phylogenic analyses of VP1 genomic region nucleotide sequences of the studied EVA71 strains of genotype C4 from Vietnam are represented in Figure 2.

The nucleotide sequences of EVA71 strains detected in HFMD and AFP patients from South Vietnam formed a monophyletic cluster with the sequences of EVA 71 strains circulated in Germany in 2018–2019 [23] and caused a large outbreak of HFMD in China in 2016–2018 [13]. The Vietnamese EVA71 strains had 99.2–99.9% of nucleotide homology with EVA71 strains which were revealed in HFMD and AFP patients studied in 2017–2018 in the Yunnan province of China.

**Figure 3:**
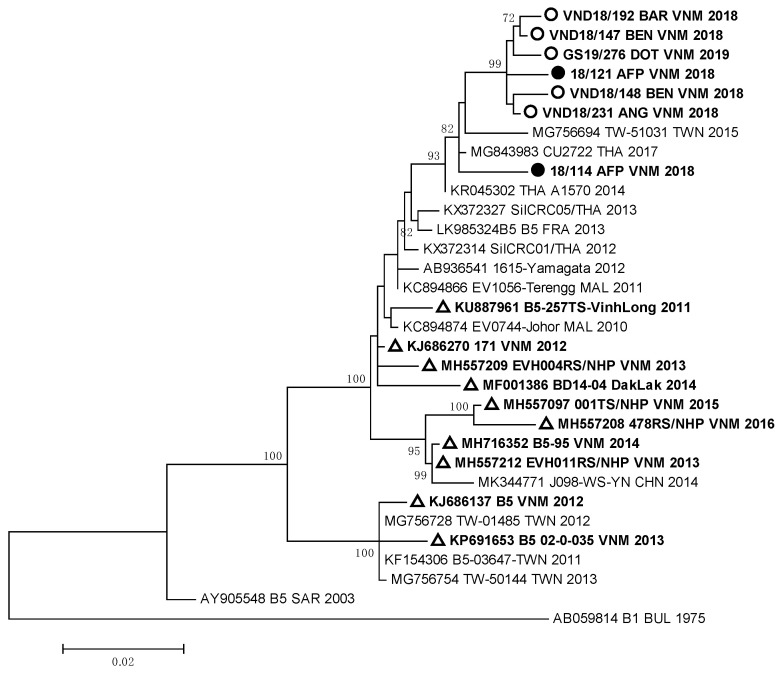
Phylogenic relationships between EVA71 strains of genotype B5 identified in Vietnam: ○—Vietnamese strains isolated from HFMD patients; ●—Vietnamese strains isolated from AFP patients; ∆—Vietnamese strains described in other studies.

Strain VND18/227 identified in one HFMD patient, who had a complicated clinical form of the disease, was very close to German EVA71 strains (99.2–99.4% of homology) of the C4 genotype. All these viruses represented a separate genetic group and had a high relationship with EVA71 strains detected in the Guangdong province of China in 2017 [12]. EVA71 viruses of the C4 genotype revealed in HFMD and AFP cases from our study were different from viruses revealed in 2003–2005 and 2011–2012 during HFMD outbreaks in South and North Vietnam [9,13,15]. They also differed from strains isolated in the Dak Lak province in 2016 [24,25]. The sequences of the studied EV-A71 strains of genotype B5 that were isolated and/or identified in HFMD and AFP patients formed a monophyletic group (Figure 3).

All B5 genotype strains showed a close relationship with Thai viruses of the B5 genotype isolated in 2017 [26], but they differed from EVA71 viruses of the B5 genotype circulated in Vietnam in 2011–2016 [25].

## 4. Discussion

The results of our research indicate the clear leadership of enterovirus 71 as the etiological agent of HFMD in South Vietnam. The prevalence of EV71 viruses among the studied strains determined the severity of HFMD in the majority of examined patients and explained multiple complications of infection. Most of the revealed HFMD cases (78%) were registered in three provinces located in the Mekong Delta. Two of them (Dong Thap and An Giang) constituted a single epidemiological focus of HFMD [27].

Our results also proved that the epidemiology of enteroviral infection in South Vietnam is affected by the fact that more than 20% of the Vietnamese population lives in the Mekong Delta [11]. The Mekong River accumulates and carries in its delta huge streams of water mixed with sewage from five countries in South-East Asia. This complicates the epidemic process and provides the highest HFMD incidence in South Vietnam. Factors (a hot and humid climate, high population density with a high share of children, and poor sanitary conditions) also support the high incidence of HFMD, as well as the widespread year-round circulation of enteroviruses among the population of South Vietnam. This contributes to the evolution of local and imported strains of enteroviruses, including EV71. This can lead to the formation of variants with altered pathogenetic properties and increased virulence [3,14,28].

It is necessary to highlight that we found a high proportion of enteroviruses 71 during the analysis of all clinical forms of enteroviral infection in South Vietnam. The EVA71 strains were isolated from all the categories of the studied patients, not only from HFMD patients but also from cases of enteroviral meningitis or meningoencephalitis. The presence of EVA71 strains in the biological material of patients who had a diagnosis of acute flaccid paralysis, including acute flaccid myelitis, was confirmed by virological and molecular methods. Among identified species A enteroviruses that were found in patients with HFMD, meningitis, or AFP, the shares of EVA71 were 50%, 54.8%, and 51.5%, respectively.

Enterovirus EVA71 clearly predominated; it is undisputable that it is the leading virus in circulation among the population in all the provinces of South Vietnam in terms of all the disease categories and case types studied. This proves the necessity to diminish the widespread circulation of EVA71 by all possible preventive measures.

The data of our molecular studies confirmed the resumption of the activity of the EVA71 virus of genotype C4 in South Vietnam, as we established that 90% of studied EVA71 strains belonged to the C4 genotype. According to phylogenic analysis, most of the EVA71 strains of the C4 genotype were a part of the same cluster together with the EVA71 strains of genotype C4 from the Chinese province of Yunnan [12]. However, these strains were different from the EVA71 strains of the C4 genotype, which circulated in Vietnam in 2003–2005 and 2011–2012. Similarly, the EVA71 strains of genotype B5 differed from the strains of the B5 genotype, which circulated in Vietnam from 2011 to 2016. They were very close to EVA71 strains of the B5 genotype identified in Thailand.

The results of the phylogenic analysis of enteroviruses indicate the influence of the largest Asian waterway, the Mekong River, the densely populated basin of which forms a unique epidemic focus of enteroviral infection, especially HFMD. In our study, we established a very close relationship between most of the Vietnamese EVA71 strains of the C4 genotype and the same EVA71 viruses circulated in the Yunnan province (China) in the upper Mekong. A close genetic relationship was shown between Vietnamese EVA71 strains of the B5 genotype and the same strains from Thailand, through which the Mekong River also flows. Thus, we determined the fact of multiple importations of EVA71 viruses from neighboring countries, and they are widespread within the country. This has led to high and sustained HFMD incidence up to the present. It is important to note that among enteroviruses isolated from AFP patients were strains belonging to both EVA71 genotypes C4 and B5. These strains did not differ genetically from strains isolated from HFMD patients.

We revealed that one case of HFMD caused by a strain (VND18/227), which genetically differed from the other EVA71 strains of the C4 genotype, was not epidemiologically associated with the other HFMD cases. This strain entered the genetic cluster together with other strains isolated in different regions of the world. Tourists could have imported this genetic variant of EVA71 into the Mekong Delta from abroad. On the other hand, it could have been introduced into South Vietnam by internal migration of the population from Central or North Vietnam. The genetic difference of 90% of EVA71 strains of genotype C4 from the strains of the same genotype that previously circulated in Vietnam confirmed the resumption of the wide circulation of the EVA71 virus of the C4 genotype in South Vietnam. The importation of new variants of EVA71 into the Mekong Delta can very likely explain the new peak in EVA71 circulation in South Vietnam.

EVA71-associated HFMD has been an emerging infection in Vietnam for many years. Our research proved the role of the EVA71 virus as the main etiological agent of HFMD, similar to the long-term studies of HFMD in southern Vietnamese provinces described in a number of scientific publications [27,28,29,30]. Co-circulation in Vietnam of EVA71 viruses belonging to two genotypes (C4, B5) described in 2018, as well as data from other publications, are in accordance with the results of our research.

The three EVA71 vaccines approved for use in China are manufactured by Chinese enterprises. These inactivated vaccines derived from the C4 genotype virus have proven efficacy against EV71-associated HFMD in children aged 6–35 months [31,32,33].

The results of a double-blinded, randomized, placebo-controlled trial of a Taiwanese inactivated vaccine, EV71vac, based on the B4 genotype virus were published [34] in 2022. The trial targeted healthy children aged 2–71 months and was done in hospitals in Taiwan and South Vietnam. The described EV71vac showed the safety, tolerability, and efficacy against EV71-associated diseases in all the participants, including infants aged 2–5 months who had the highest case severity and fatality rate. EV71vac has the capacity to protect against B4, B5, and C4 genotypes for at least 1 year. Previously EV71vac in vitro cross-reaction against B5, C4, and C5 genotypes was shown [35]. EV71vac continued to protect children in the vaccine group when from the end of 2020 to April 2021, Vietnam had an EV71 outbreak. Laboratory-confirmed cases of EVA71-associated HFMD were fixed only in the placebo group, in which 22 patients were infected with B5 and C4 genotype viruses. The results of this trial have been submitted to the regulatory authorities in Taiwan and Vietnam. Once approved, this non-polio enterovirus vaccine (EV71vac) will be ready for distribution [34,35,36].

HFMD is the most common form of enteroviral infection in South-East Asia. Over the past 25 years, the region has had numerous HFMD outbreaks with severe clinical manifestations mainly connected with EVA71 in Taiwan, China, and Vietnam (mostly South Vietnam) [12,14,15,26,37]. The disease often had multiple complications. It could even be fatal, mainly in infants and children, whose lethality ranged from 0.5% to 19% in different years [14].

Therefore, we showed that in South Vietnam, the main etiological agent of HFMD and other enteroviral infections (including AFP) was EVA71 (predominantly genotypes C4 and B5). While EVA71 strains that caused acute flaccid paralysis did not differ genetically from strains isolated from patients with HFMD, it may be concluded that vaccination against EVA71 would affect both the morbidity rates of HFMD and AFP. The results of our study provide important information on the circulation of HFMD etiological agents in the country. They help to understand patterns within the evolution of the epidemic process as well as other forms of enteroviral infection.

The obtained data once again emphasizes that active epidemiological and virological surveillance plays a key role in informing the public health authorities about the real epidemic situation in order to take appropriate measures. The main goal of complex anti-epidemic measures is to diminish the incidence of HFMD and the economic burden of this infection in all possible ways. For improving epidemiological surveillance, it is necessary to analyze the long-term incidence of HFMD and other EVI, along with an assessment of the influence of risk factors and the main causes of outbreak emergence.

Collaborative scientific research aimed at studying the epidemiology and properties of pathogens is a necessary condition for preventing the risks associated with enteroviral infection. In order to improve epidemiological surveillance and increase its effectiveness, it is necessary to establish systematic monitoring of enterovirus circulation, paying special attention to the spread of new virus variants. This will make it possible to foresee the development of outbreaks and to take timely preventive measures, based on new technological solutions, especially in the field of vaccination. Improvement of epidemiological surveillance should be combined with simultaneous improvement of laboratory diagnostics based on the implementation of new virological and molecular methods.

## 5. Conclusions

HFMD is an extremely important public health problem in Vietnam. Since the beginning of the 21st century, outbreaks of HFMD (mainly associated with enterovirus EVA71) have been cyclically registered all over Vietnam. Systematic monitoring of the circulation of EVA71 and other enteroviruses performed in the country is a means to trace viral evolution, including the formation and spread of novel enterovirus variants, predict changes in the epidemic situation, and develop adequate prevention strategies. The need to reduce the economic and social damage caused by HFMD in Vietnam requires increased HFMD prevention effectiveness and improved vaccination policies. Proper implementation of vaccination, an effective and reliable means for the prevention of infectious diseases, has saved mankind from outbreaks and mortality caused by numerous severe infections. Vaccine-based protection from non-polio enteroviral infection in Vietnam and implementation of vaccination against EVA71-associated infection are indispensable. The EV71 vaccines, which exist and are produced in Southeast Asia, can serve as a good tool to combat HFMD. They will help to solve the problem of enterovirus 71-associated infection so important for Vietnam.

## Figures and Tables

**Table 1:** Epidemiological and clinical data of patients with hand-foot-and-mouth disease involved in the study.

Characteristics	Number of Patients	%
Age		
0–12 months	19	21.8
13–35 months	58	66.7
3–6 years	10	11.5
Gender		
Male	54	62.1
Female	33	37.9
Severity Mild clinical form (buccal ulcer, skin rash) Complicated clinical form (buccal ulcer, skin rash, neurological, cardiovascular, respiratory, and paralytic symptoms)		
	12	13.8
	
	75	86.2
Total	87	100

**Table 2:** Epidemiological data of patients with acute flaccid paralysis involved in the study.

Characteristics	Number of Patients	%
Age		
0–12 months	1	3.8
13–35 months	17	65.4
3–14 years	8	30.8
Gender		
Male	17	65.4
Female	9	34.6
Vaccination		
Vaccinated (3 OPV doses)	19	73.1
Non-vaccinated	7	26.9
Total	26	100

## Data Availability

Data will be available on reasonable request to the corresponding author.
